# IFITM proteins inhibit the late step of feline foamy virus replication

**DOI:** 10.1080/19768354.2020.1819413

**Published:** 2020-09-15

**Authors:** Jinsun Kim, Cha-Gyun Shin

**Affiliations:** Department of Systems Biotechnology, Chung-Ang University, Ansung, Republic of Korea

**Keywords:** IFITM, feline foamy virus, late step

## Abstract

Interferon-induced transmembrane (IFITM) proteins as host restriction factors are known to inhibit the replication of several viruses. In this study, transient IFITM expression vectors were used to investigate whether IFITMs inhibit feline foamy viral (FFV) replication and which step of viral replication is inhibited. In our studies, viral production was significantly reduced when cells were infected with FFV at almost same times such as −3, 0, or 3 h post-transfection with IFITM vector. However viral production was not reduced even though cells were infected with FFV at 3 or 6 days post-transfection when production of IFITM proteins was maximized. Considering that IFITM expression was maximized at 3 days post-transfection, the stage of viral replication inhibited by IFITM appears to be the late step of viral replication. Moreover, the viral Gag proteins detected in the virus-infected cell lysates were proportionally correlated with viral titer of the culture supernatants. Therefore, it is likely that IFITMs can restrict production of FFV at the late step of viral replication.

## 1. Introduction

Host restriction factors are the cellular proteins associated with the function of the host innate immune systems against the infecting viruses (Lilly 1970). Those proteins are frequently induced by interferons, which establish dynamic host defense mechanisms through the induction of the expression of various genes that encode many antiviral innate immune effectors. The interferon-stimulated genes (ISGs) encode for a well-known group of proteins that have wide range of functions, including cell growth and metabolism, in host cell systems (Narayana et al. 2015). Among ISGs, antiviral factors have been studied extensively in the field of virus research. At present, several host restriction factors have been reported to have antiviral effects at a specific step of viral replication cycle. For example, SAMHD1 is a known restriction factor that limits retroviral replication at the reverse transcription step (Ballana and Esté 2015).

Interferon-induced transmembrane (IFITM) proteins are a family of interferon-induced antiviral proteins (Narayana et al. 2015). IFITM proteins restrict a broad range of viral replication, including that of the influenza virus, dengue virus, filoviruses, coronavirus, hepatitis C virus, lyssaviruses, orthomyxovirus, flavivirus, and West Nile virus (Xie et al. 2015). However, some viruses, such as human papillomavirus, human cytomegalovirus, Machupo virus, mouse leukemia virus, Lassa virus, lymphocytic choriomeningitis virus, murine leukemia virus, and foot-and-mouth disease virus, are unaffected or only slightly affected by IFITM proteins (Xie et al. 2015). IFITM proteins are found in a wide range of species, from amphibians, fish, and birds to mammals (Muñoz-Moreno et al. 2016). There are five IFITM genes such as IFITM 1, 2, 3, 5, and 10 in humans (Lee et al. 2018). The expression of IFITM 1, 2, and 3 is stimulated by type 1 and 2 interferons owing to the presence of the interferon stimulation response element in their promoters, whereas IFITM 5 and 10 are not induced by interferon (Jia et al. 2015). Owing to its involvement in bone mineralization and maturation, IFITM 5 is primarily expressed in osteoblasts. The function of IFITM 10 remains unclear. IFITM 1, 2, and 3 are expressed in a variety of tissues. IFITM 2 and IFITM 3 are mainly found in late endosomes and early endosomes, respectively (Smith et al. 2019), and IFITM 1 is expressed primarily on the plasma membrane (Gorman et al. 2016).

Foamy viruses (FVs) are a genus of the subfamily Spumaretrovirinae, belonging to Retroviridae family (Coffin et al. 1997). FVs have been found in some mammalian hosts such as non-human primates (prototype foamy virus), cattle (bovine foamy virus), cats (feline foamy virus), and horses (equine foamy virus) (Coffin et al. 1997). Recently, feline foamy virus (FFV) has been studied extensively to understand viral replication and life cycle to provide essential information to develop FFV-derived viral vectors for gene therapy (Lee et al. 2019). In this work, we investigated whether IFITMs can inhibit FFV replication and determine which step of viral replication is blocked.

## 2. Materials and methods

### 2.1. Cell culture

The feline kidney cell line, CRFK (Crandell-Ress feline kidney, Korean Cell Line Bank, Seoul, Korea) was grown and maintained in Dulbecco’s modified Eagle’s Medium (DMEM) supplemented with 2 mM L-glutamine, 10% heat-inactivated fetal bovine serum (Sigma-Aldrich, St. Louis, MO, USA), 100 U/mL penicillin, and 100 μg/mL streptomycin. The FeFAB cell lines derived from CRFK cell line containing FFV LTR-β-galactosidase reporter cassettes were maintained by adding 600μg/mL G418 to DMEM (Löchelt et al. 1993). All cultures were incubated at 37°C in 5% CO_2_.

### 2.2. IFITM expression vectors preparation

Human IFITM 1, 2, 3 complete DNAs were obtained from Addgene (plasmid no. 58399, 58398, 58397, respectively). For powerful transient expression, these DNAs were inserted into the pEGFP-C3 vector in which the EGFP gene was deleted. For this insertion, each IFITM gene was amplified by PCR using DNA oligo primer (Supplementary Table 1). All PCR reactions were performed with *Pfu* DNA polymerase (Bioneer, Korea) to prevent DNA synthesis errors. Briefly, the IFITM 1 gene was amplified with following two primers, IF1-1S-Age1 and IF1-125A-BamH1. Amplified IFITM 1 DNA was digested with Age1 and BamH1 and ligated with digested pEGFP(-)-C3 with same enzymes. IFITM 2 and 3 DNA were ligated in the same manner. The sequences of PCR-amplified DNA fragments were verified with restriction enzyme analysis and by the commercial sequencing company (Solgent, Korea).

### 2.3. IFITM expression test

Successfully cloned IFITM 1, 2, and 3 expression vectors were transfected into CRFK cell line by polyethylenimine (PEI) method to confirm transient expression. In all transfection experiments, 2.6 µg of expression vector was used. Transfected CRFK cells were collected at 12 h, 1, 2, 3, 4, 5, 6, and 7 days post-transfection, lysed in RIPA buffer [50 mM Tris-HCl (pH 7.4), 150 mM NaCl, 0.1% SDS, 0.5% (w/v) sodium deoxycholate], and centrifuged at 22,000 *g* for 15 min at 4°C. After centrifugation, the clarified lysates were collected, and stored at −20°C for later usage. Expression of IFITM1, IFITM2, and IFITM3 in all samples was confirmed by western blot with antibodies to IFITMs described below.

### 2.4. Viral production with transient IFITMs expression

First, 2 × 10^5^ CRFK cells were grown in DMEM supplemented with 10% FBS in 6-well plates for 1 d prior to transfection. In all transfection experiments, 2.6 µg of IFITM expression vector was transfected by PEI. At 3 h post-transfection, the culture supernatant was discarded, and new medium was added. In all infection experiments, the cells were infected with FFV with a MOI 1. At 3 h post-infection, the medium was exchanged for fresh medium. Each FFV infection experiment was conducted separately, with non-transfected CRFK cells used as the positive control for viral infection. In the protocols 1–4, the culture supernatants and cell lysates were prepared at 3 days post-infection. In the protocol 5, they were prepared at 75 h post-infection. Each supernatant was harvested by centrifuging in 22,000 g for 15 min at 4°C. The supernatant was collected and stored at −70°C for later use. The collected supernatants were used to infect FeFAB cells to determine the viral titer. Cell lysate was collected as described above.

### 2.5. FeFAB assay

FeFAB cells (a kind gift from Dr Martin Löchelt, Germany) are modified CRFK cells that are used for FFV infection experiments. FeFAB cells express β-galactosidase under the control of the FFV LTR, which is trans-activated by the FFV Tas protein in relation to the level of virus replication. Approximately 2×10^4^ FeFAB cells were infected with serially diluted supernatants. After 48 hr, the cells were fixed with fixer solution (0.2% (w/v) glutaraldehyde, 1% (w/v) formaldehyde in PBS). After washing with PBS, the fixed FeFAB cells were incubated for 4 hr with an X-Gal (5-bromo-4-chloro-3-indolyl-β-D-galactopyranoside) staining solution. The number of blue cells was then counted using an inverted microscope. All data are representative of three independent experiments with triplicate samples.

### 2.6. Western blot analysis

In all experiments, equal amounts of cell lysates were blotted with in-house rabbit polyclonal antibody against FFV-gag (1:500 dilution), mouse monoclonal antibody against IFITM 1 (1:500 dilution), and rabbit monoclonal antibodies against IFITM 2 (1:2000 dilution), and 3 (1:2000 dilution). The cell lysates were probed with mouse monoclonal antibody against β-actin (1:5000 dilution, Thermo Scientific, Waltham, MA, USA) as an internal control.

### 2.7. Statistical analysis

Data are expressed as the mean ± SEM from the triplicates of the three independent experiments. Statistical significance was analyzed with a two-paired Student's *t*-test. * = *p* < 0.05, ** = *p* < 0.01, *** = *p* < 0.001.

## 3. Results

### 3.1. Expression patterns of IFITMs

**Figure 1.**
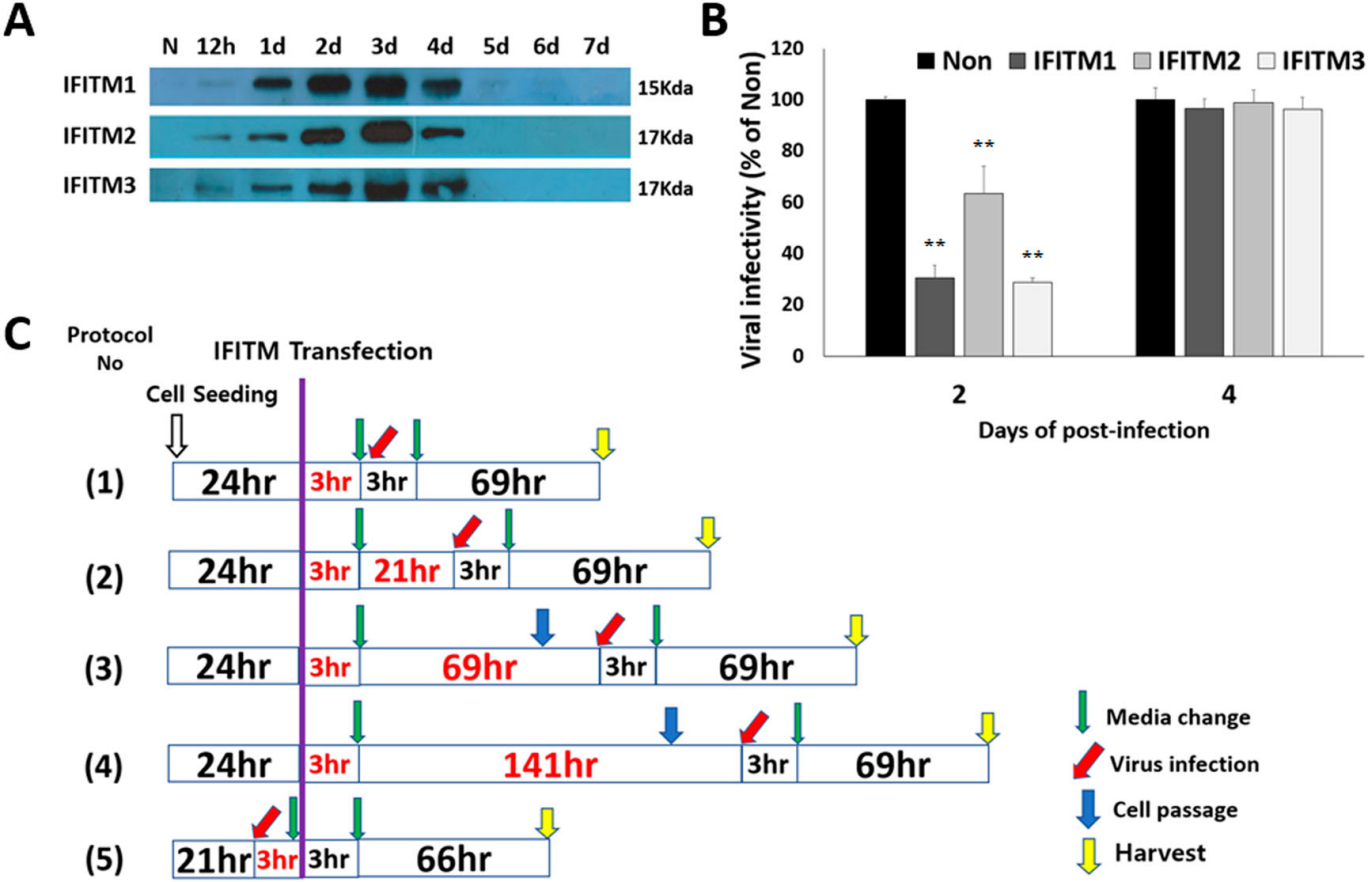
Scheme of FFV infection experiments. (A) Transient expression patterns of IFITMs. In 6-well plate, 2 × 10^5^ CRFK cells were grown for 1 day before transfection. On the day of transfection, 2.6 µg of the IFITM 1, 2, 3 expression vectors were transfected by PEI. At the indicated post-transfection days, cell lysates were harvested by centrifuging at 22,000 *g* for 15 min at 4°C. The collected cell lysates were stored at –20°C for later usage. Expression of IFITM1, IFITM2, and IFITM3 in all samples was confirmed by western blotting with antibodies to IFITM1 (Santa Cruz), IFITM2 (Proteintech), and IFITM3 (Abcam). (B) Initial experiments of FFV and IFITMs. In 6-well plate, 2×10^5^ CRFK cells were grown for 1 day before transfection. 2.6 µg of the IFITM 1, 2, 3 expression vectors were transfected by PEI on the next day. FFV was infected at 2 days post-transfection (MOI 1.0). The supernatants were collected at 2, 4 post-infection days, and the virus titers were measured by FeFAB assay. C. Five different protocols of FFV infection experiments. In 6-well plates, 2 × 10^5^ CRFK cells were grown for 1 day before transfection. On the day of transfection, 2.6 µg of the IFITM 1, 2, 3 expression vectors were transfected by PEI. After 3 h, the medium was exchanged for fresh medium (green arrow). FFV infection took place at 3 h after (1), 1 day after (2), 3 days after (3), 6 days after (4), and 3 h before (5) transfection. Green arrows indicate a change in medium (3 h). Red arrows indicate virus infection (MOI 1.0). Blue arrows indicate cell passaging, to prevent cell accumulation and death.

In order to know when IFITM proteins are produced at maximum level on transfection in CRFK cells, IFITM expression vectors were transfected by PEI, respectively. At 0.5, 1, 2, 3, 4, 5, 6 and 7 post-transfection days, cell lysates were prepared, respectively, and the IFITM proteins were detected by western blotting. The amounts of IFITM proteins increased gradually from 12 h to 3 days post-transfection, but after 4 days post-transfection those of IFITM proteins decreased. The bands of the proteins were not detected on the blot from 6 days post-transfection (Figure 1(A)). Therefore it was clear that IFITM proteins were produced at maximum on 3 days post-transfection.

### 3.2. Effect of IFITMs on FFV replication

To understand the effect of IFITM proteins on FFV replication, initially, CRFK cells transfected with IFITM DNA were infected with FFV at 2 days of post-transfection. Then the culture supernatants were collected at the 2 days of post-infection. The infectious viral titer of the supernatants was evaluated by FeFAB assay.

It was found that the FFV viral titers of the IFITM-transfected samples were significantly lower than that of the non-transfected control at 2 days post-infection (4 days post-transfection) (Figure 1(B)). However, the viral titers were rescued to almost same level with that of the non-transfected control at 4 days post-infection (6 days post-infection). The results indicated that the IFITM expression could suppress viral replication in the infected cells.

To determine which step of the viral life cycle is affected by IFITM proteins, five different experiment protocols were designed, considering that IFITM expressions are maximized at 3 days post-transfection (Figure 1(C)). In the four protocols, viral infection started at 3 h (Protocol 1), 1 d (Protocol 2), 3 days (Protocol 3), or 6 days (Protocol 4) after IFITM transfection, respectively. In Protocol 5, viral infection started at 3 h before IFITM transfection. In all the protocols culture supernatants were collected to evaluate viral titers at 3 days post-infection, and then cells were lysed to detect viral Gag protein and IFITM proteins.

**Figure 2.**
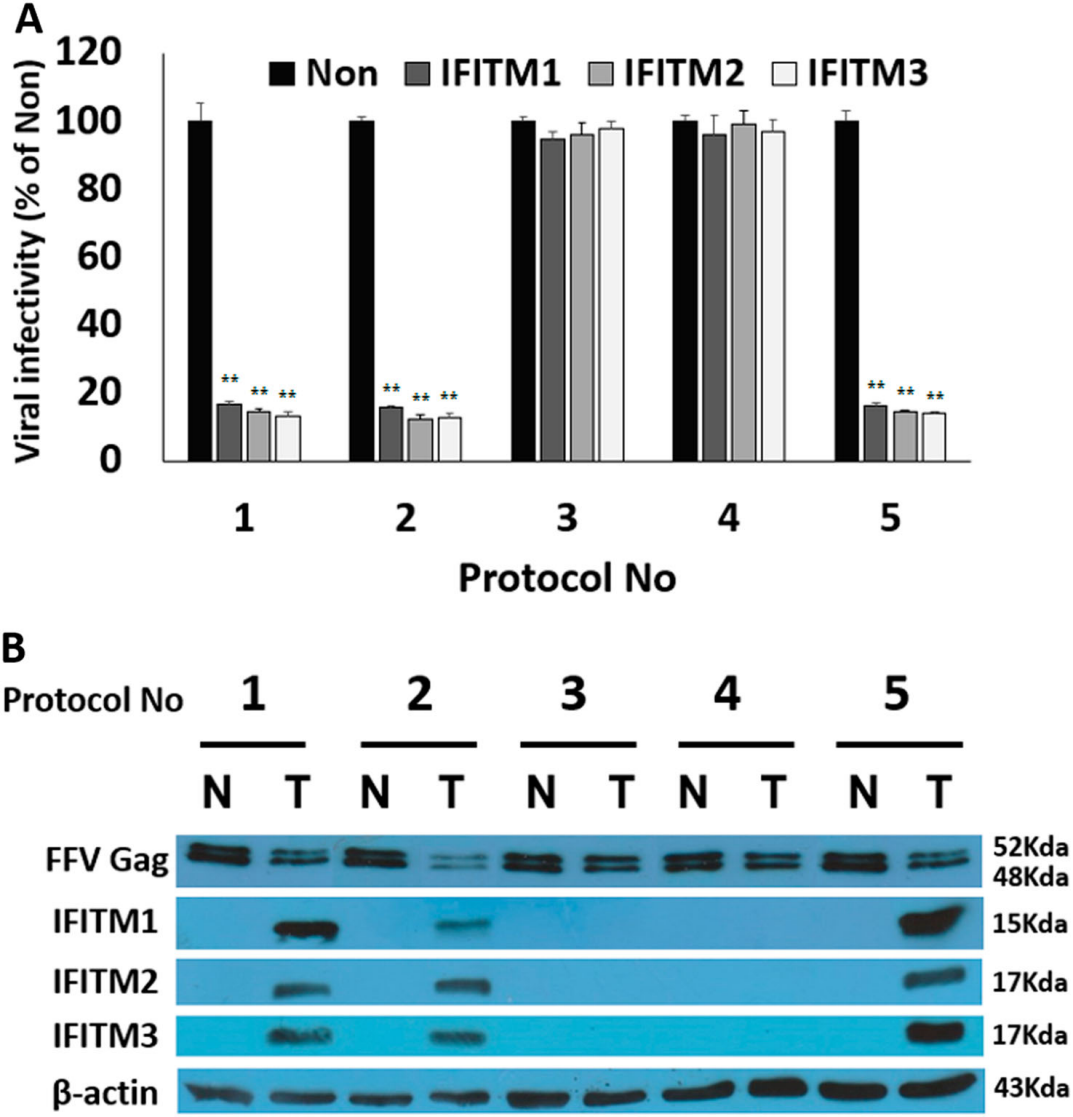
Human IFITMs inhibit foamy viral replication at the late step. At 3 days post-infection, every supernatant and cell lysates were harvested by centrifuging in 22,000 *g* for 15 min at 4°C. The supernatants were collected and stored at –70°C for later use. The collected cell lysates were also stored at –20°C for later usage. (A) Viral titers of each sample. Viral titers for measurement of infectious viruses present in the supernatants were evaluated by using the FeFAB assay. The results were presented as percentages of the non-transfected sample. All data are representative of three independent experiments performed in triplicate. (B) Confirmation of FFV Gag protein and IFITM proteins in cell lysates. Expression of FFV Gag protein and IFITM proteins in all the samples was confirmed by western blotting with FFV-Gag specific polyclonal antibody, IFITM1 (Santa Cruz), IFITM2 (Proteintech), and IFITM3 (Abcam) antibodies; β-actin (Santa Cruz) was used as the loading control. Data are expressed as the mean ± SEM from the triplicates of the three independent experiments. Statistical significance was analyzed with a two-paired Student’s *t*-test. * *p *<* *.05, **p <* *.01.

Viral titers of the IFITM transfected cells were expressed as % of non-transfected control prepared by the same protocol. The results are shown in Figure 2(A). As expected, a significant reduction in viral titers was observed in Protocols 1, 2, and 5 where the viral infection started at 3 h post-transfection, 1 d post-transfection, and 3 h pre-transfection, respectively (Figure 2(A), Graphs 1, 2, and 5; Supplementary Fig. S1). In these cases, all IFITM transfection samples were found to have only 10–20% of the viral titer of the non-transfected control. With regarding to inhibitory strength of viral production, there were no significant differences among IFITM 1, 2, and 3. Therefore it appears that IFITM 1, 2, and 3 may repress viral production at the same step of viral replication through a similar mechanism. However, when viral infection occurred at 3 and 6 days post-transfection (Protocols 3 and 4, respectively), viral production was not inhibited as the viral titers were very similar to those of non-transfected samples (Figure 2(A), Graphs 3 and 4; Supplementary Fig. S1).

In order to re-confirm viral titers shown in Figure 2(A) with viral protein, the levels of IFITM proteins and viral Gag protein in viral lysates were investigated (Figure 2(B)). IFITM proteins were observed in the cell lysates that were prepared at 3, and 4 days post-transfection (Figure 2(B), T lines in Protocols 1, 2, and 5), indicating that IFITM expression is maximized at approximately 3 days post-transfection. However the cell lysates prepared at 6 days or 9 days post-transfection did not contain IFITM proteins (Figure 2(B), T lines in Protocols 3 and 4). Next, viral Gag proteins were investigated in all the samples. When the IFITM 1-transfected samples were investigated for the FFV Gag protein, it was observed relatively in small amounts in cell lysates of the 3 or 4 days post-transfection (Figure 2(B), T lines in Protocols 1, 2, and 5), compared with the non-transfected sample (Figure 2(B), N lines in Protocols 1, 2, and 5). However, very significant amounts of FFV Gag protein were found in the cell lysates of the 6 or 9 days post-transfection (Figure 2(B), T lines in Protocols 3 and 4), compared with the non-transfected sample (Figure 2(B), N lines in Protocols 3 and 4). In addition, very similar results for FFV Gag protein were observed in the IFITM 2-transfected samples and in the IFITM3-transfected samples [Unpublished observations]. The viral titers, detected as blue cells in the FeFAB assay, are shown in Supplementary Fig. S1.

## 4. Discussion

Most of the viruses blocked by IFITMs are enveloped RNA viruses, which enter cells through clathrin-mediated endocytosis. Inhibition usually occurs during the entry step, especially during the fusion process between the virus and the endosomal membrane (Huang et al. 2011; Ranjbar et al. 2015; Xie et al. 2015; Wang et al. 2017). IFITMs appear to inhibit membrane fusion between the viral envelope and the cellular membranes, such as the endosomal membrane, the plasma membrane, and the exosomal membrane. Therefore, depending on the virus, it is possible that the presence of IFITM on the membrane disturbs the formation of viral vesicles in the late step of viral replication, such as the specific interaction between the viral capsid and viral envelope proteins.

In these studies, the results are suggesting two facts. First, IFITM proteins are efficiently inhibiting FFV replication when their expression is maximized at approximately 3 days post-transfection. Second, IFITM proteins inhibit FFV replication at the late step rather than at the entry step. In Protocol 3, the expression of IFITM proteins was maximum when the cells were infected with FFV. However, the viral titer was very similar to that of non-transfected control. Therefore, this result supports that IFITM proteins do not block FFV infection at the early infection step.

**Figure 3.**
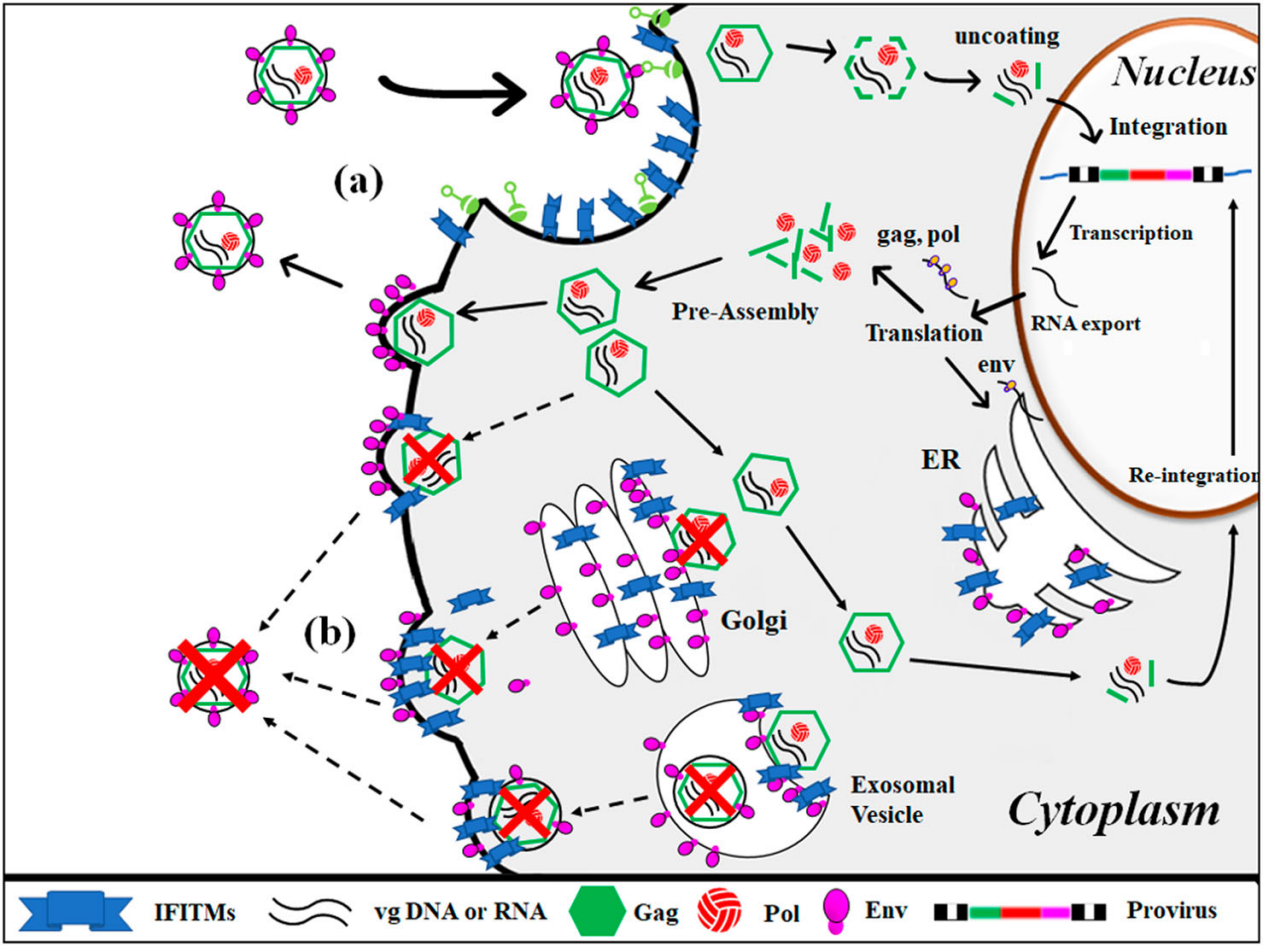
IFITM is proposed to repress FFV production by disturbing viral assembly at the intracellular and plasma membranes. (a) At the early step (viral entry), IFITM present on the plasma membrane does not efficiently inhibit viral infection. (b) At the late step (viral assembly and release), IFITM present on the intracellular and plasma membranes efficiently inhibits viral assembly by blocking the interaction between pre-assembled capsid and envelope proteins present on membranes. Data are expressed as the mean ± SEM from the triplicates of the three independent experiments. Statistical significance was analyzed with a two-paired Student's *t*-test. *p <* *.05, **p <* *.01.

In contrast, the viral titers were significantly reduced when cells were infected with FFV and transfected with IFITM DNAs at almost same times, which was done according to Protocol 1, 2, and 5, respectively. In these condition the levels of IFITM proteins were maximum as approximately 3, or 4 days post-transfection when the infected cells were lysed at the 3 days post-infection. Therefore, it is proposed that human IFITM 1, 2, and 3 inhibits viral production more efficiently at the late step rather than at the early infection step of the foamy viral replication cycle (Figure 3).

As foamy viral assembly occurs at intracellular membranes as well as at the plasma membrane (Lindermann and Rethwilm 2011; Hamann and Lindermann 2016), it is suggested that IFITMs as membrane proteins may disturb viral assembly at the intracellular membrane and plasma membrane by interacting viral Gag proteins. FFV Gag proteins or capsid structures, which already interacted with IFITM proteins, can’t further interact with viral envelope protein present on intracellular membranes, which results in inhibition of successful viral assembly.

Currently, we are designing more detailed experiments to study the interaction between FFV Gag protein and IFITM 1, 2, 3 proteins. If IFITM proteins as membrane proteins interact with FFV Gag protein with higher affinity, compared to FFV envelope protein, our suggestion will be really confirmed.

## Supplementary Material

Supplemental Material
