# Supplementary material for: IFITM proteins inhibit the late step of feline foamy virus replication
Source: Anim Cells Syst (Seoul). 2020 Sep 15;24(5):282–8. doi: 10.1080/19768354.2020.1819413 (PMC7646556; doi:10.1080/19768354.2020.1819413)

Supplementary Table 1. Oligonucleotide primers used for this study

| Name | Sequence | Aim |
| --- | --- | --- |
| IF1-1S-Age1 | 5'-CGCTA CCGGT CGCCA CCATG GTGCA CAAGG  AGGAA CATGA G-3' | Human IFITM1 Cloning |
| IF1-125A-BamH1 | 5'-TGGGG GATCC CTAGT AACCC CGTTT TTCCT G-3' | Human IFITM1 Cloning |
| IF2-1S-Age1 | 5'-CGCTA CCGGT CGCCA CCATG GTGAA CCACA  TTGTG CAAAC C-3' | Human IFITM2 Cloning |
| IF2-132A-BamH1 | 5'-GCCTG GATCC CTATC GCTGG GCCTG GACGA C-3' | Human IFITM2 Cloning |
| IF3-1S-Age1 | 5'-CGCTA CCGGT CGCCA CCATG GTGAA TCACA  CTGTC CAAAC C-3' | Human IFITM3 Cloning |
| IF3-133A-BamH1 | 5'-GCCTG GATCC CTATC CATAG GCCTG GAAGA TC-3' | Human IFITM3 Cloning |

Underline is to indicate restriction sites.


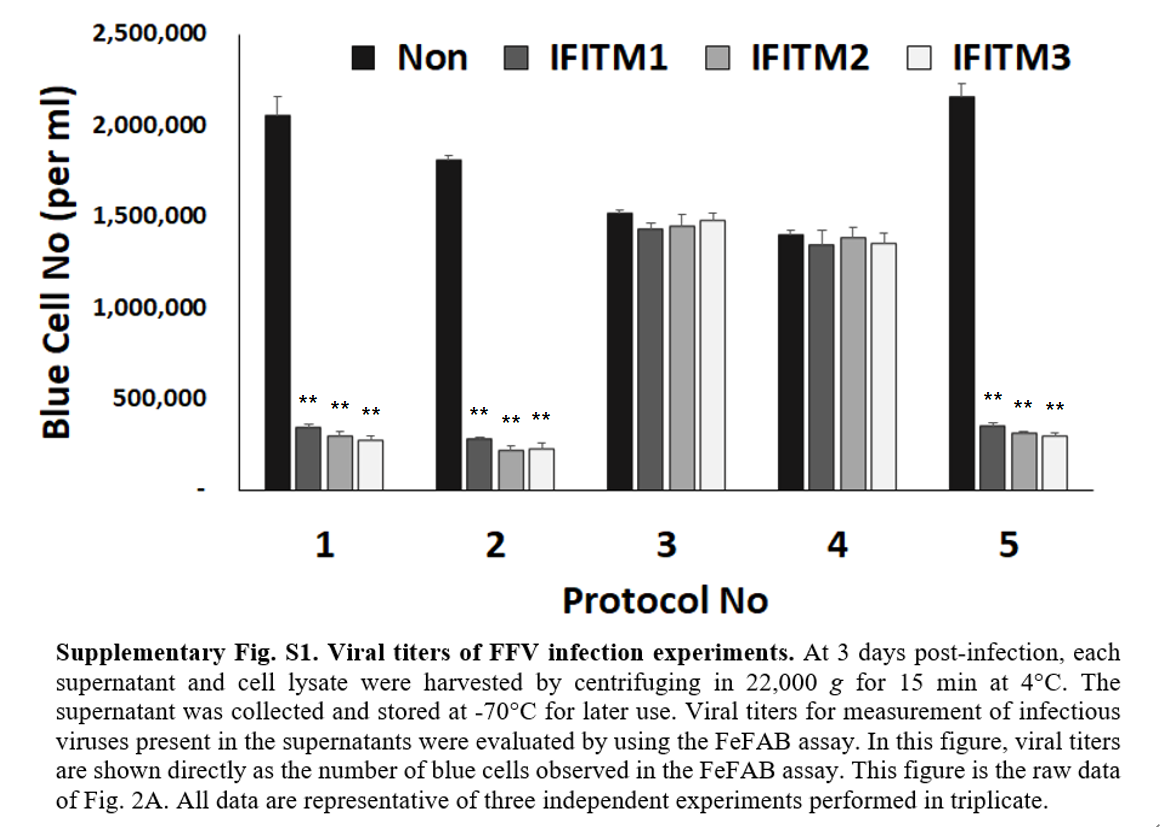

Supplement: Supplemental Material [file TACS_A_1819413_SM2881.docx]
